# Anesthesia strategies for elderly patients with craniocerebral injury due to foreign-body penetration in the plateau region: a case report

**DOI:** 10.3389/fmed.2024.1385603

**Published:** 2024-05-13

**Authors:** Yongtao Sun, Yang Liu, Peng Liu, Min Zhang, Mengjie Liu, Yuelan Wang

**Affiliations:** ^1^Department of Anesthesiology, The First Affiliated Hospital of Shandong First Medical University & Shandong Provincial Qianfoshan Hospital, Shandong Institute of Anesthesia and Respiratory Critical Medicine, Jinan, China; ^2^Shandong First Medical University & Shandong Academy of Medical Sciences, Jinan, China; ^3^Department of Anesthesiology, Shandong Provincial Hospital Affiliated to Shandong First Medical University (Shandong Provincial Hospital), Jinan, China

**Keywords:** plateau area, elderly, foreign-body penetration, craniocerebral injury, anesthesia strategies, long-term

## Abstract

**Background:**

The administration of anesthesia for elderly individuals who are critically ill, suffering from severe craniocerebral injuries, and living in plateau regions presents a rare, intricate, and high-risk challenge. This case study outlines the specific anesthesia management protocols necessary for plateau-dwelling patients with significant craniocerebral damage undergoing prolonged invasive procedures.

**Case report:**

A 76-year-old male patient had a 26-year history of foreign-body penetration of the skull and had experienced local purulent discharge and pain for the previous 20 days. The diagnoses included right hypoplasia, a foreign body in the skull with an infection, hypokalemia, hypoproteinemia, pulmonary fibrous foci, and bilateral pleural effusion. For almost 6 months, the patient suffered from recurring headaches, blurred vision, and sluggish bodily movement. The patient had a poor diet, poor sleep quality, normal urination, and no noticeable weight loss since the onset of the illness. The right anterior ear had a 2 cm skin abscess with yellow pus and a black metal foreign body tip. The left eyelid was red and swollen, and the left conjunctiva was hyperemic; the right eyelid showed no abnormalities, and both pupils were wide and round, with light and adjustment reflexes and no cyanosis on the lips. Skull development was normal. No dry or moist rales were audible in either lung. The heart rhythm was regular, and the heart rate was 50 bpm. Chest CT revealed left lung calcification foci, bilateral pleural effusion, and fiber foci in the lower lobes of both lungs.

**Conclusion:**

Furthermore, the patient in question was of advanced age and had a complex medical history, including prolonged exposure to high altitudes and previous instances of severe craniocerebral trauma, among other uncommon pathophysiological characteristics. In particular, the patient also underwent surgical interventions at both high and low altitudes, adding to the complexity of their case. To ensure patient safety, close multidisciplinary collaboration, the development of a precise surgical plan, and the implementation of a suitable perioperative anesthetic management strategy are imperative.

## Introduction

Residents of high-altitude regions are subject to chronic hypoxia, resulting in prolonged exposure to a low-oxygen environment that initiates a series of physiological changes. These changes encompass increased production of red blood cells, elevated levels of hematocrit, higher blood viscosity, decreased oxygen saturation in the blood, greater capillary density, slower blood flow in microcirculation, enhanced capillary permeability, increased vulnerability to bleeding and clotting, and the development of microthrombi (1). Several studies have demonstrated an increase in factors contributing to Virchow’s triad (hypercoagulability, venous stasis, and vessel wall injury) at high altitudes (2, 3). Many ailments affecting patients in the plateau area have not been treated promptly due to long-term hypoxia, changing lifestyle habits, stigmatization of ethnic and cultural beliefs (craniotomy is stigmatized), and limitations in medical care. Patients with severe craniocerebral damage caused by foreign-body penetration have a significant risk of bleeding and infection (4, 5). To ensure the safety of such patients, appropriate surgical treatment and precise anesthesia administration are essential. This study details a rare case of a patient with a 26-year history of a foreign body penetrating the brain, which could have resulted in a brain abscess, subsequent disease, or even death.

Patients residing at high altitudes exhibit a higher prevalence of comorbidities, necessitating the proactive involvement of a multidisciplinary team (MDT) in the evaluation of surgical, anesthetic, and postoperative care plans. While pre-anesthetic medication protocols remain largely unchanged from those in low-altitude regions, heightened levels of stress, anxiety, and apprehension may elevate oxygen consumption, prompting the judicious use of sedatives as deemed necessary. A comprehensive evaluation is imperative prior to emergency surgeries with a high level of risk, including thoracoabdominal injuries, brain injuries in conjunction with other injuries, and other critical emergency procedures. Of particular concern are elderly individuals residing at high altitudes, as they are susceptible to the effects of prolonged hypoxia exposure, resulting in diminished physiological reserves and decreased capacity to withstand the stresses of anesthesia and surgery. Medications should be limited in variety, administered in small doses, and given in divided doses to mitigate potential complications. Surgeons must assess the risks for such patients, including the need for surgery at a low altitude for high-altitude patients and the need for accurate removal of intracranial foreign bodies without damaging the surrounding organs. The potential for massive intraoperative bleeding, septic shock, systemic inflammatory reactions, hyperlactatemia, and emergency rescue increases the difficulty of surgery and anesthesia management (6, 7). The literature describing anesthesia administration in patients undergoing such surgery is limited because information on long-term, invasive surgery for severe craniocerebral injury is rare.

## Case report

A 76-year-old man with a height of 163 cm and weight of 65 kg complained of a cranial foreign body for 26 years and local purulent discharge and pain for 20 days. The patient sustained head and facial trauma 26 years ago after collapsing following an altercation with others. Due to the limited medical resources and absence of radiation equipment during that period, the patient received only debridement and suturing. Furthermore, due to the patient’s favorable postoperative recovery and financial constraints, no additional medical interventions were pursued. An X-ray obtained 8 years ago revealed a knife-shaped foreign object inside his skull causing persistent purulent discharge from his right cheek. He had experienced frequent headaches, blurred vision, and limb weakness over the past 6 months. Because the local hospital was limited in providing care for certain medical conditions, he was transferred to the Department of Otorhinolaryngology in our hospital on 26 March 2020. The patient underwent surgery for intestinal obstruction 20 years prior and had no preexisting conditions. This case report was approved by the hospital ethics committee, and an informed consent was obtained from the patient.

**Figure 1:**
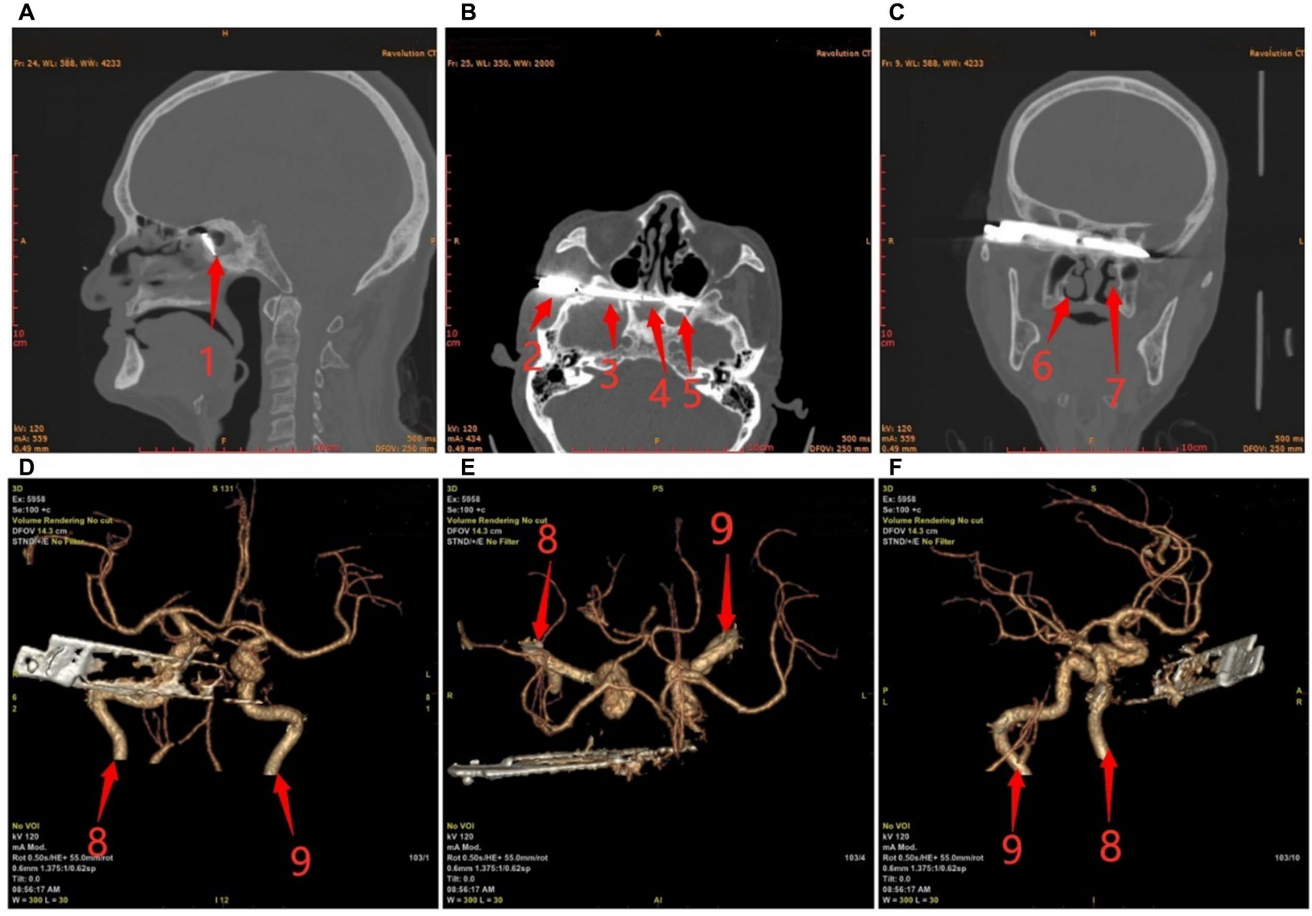
Preoperative craniocerebral CT and reconstruction. **(A)** Sagittal; **(B)** Transverse; **(C)** Coronal; **(D–F)** The positional relationship between blood vessels and knives. Red arrows: 1, sphenoid sinus; 2, temporal fossae; 3, the base of the middle cranial fossa; 4, sphenoid sinuses (right); 5, sphenoid sinus (left); 6, right nasal cavity; 7, left nasal cavity; 8, right internal carotid artery; and 9, left internal carotid artery.

Preoperative ECG and 24 h dynamic electrocardiogram showed sinus bradycardia with 35–50 BPM and multi-lead ST-T alterations. A chest CT revealed bilateral pleural effusion and calcification in the left lung. There were metal foreign bodies in the right temporal and orbital regions, discontinuous bone in the right temporal bone, orbital wall, and sphenoid sinus, and involvement of the right optic nerve, according to the results of paranasal sinus CT, cranial neck CTA, and skull reconstruction (Figure 1).

On April 2, 2020, the foreign bodies were removed from the compound operating room. Before surgery, multidisciplinary consultations were held in the departments of otorhinolaryngology, anesthesia, cardiology, neurology, neurosurgery, CT imaging, etc., to enhance preoperative evaluation, treat hypoproteinemia, and manage infection.

After entering the compound operating room, BP, HR, SpO_2_, ECG, and BIS were routinely monitored, an intravenous line was secured in the left upper limb, and an invasive arterial line was secured in the right radial artery under local anesthesia. Following the verification of unimpeded mask ventilation, oxygen denitrogenation was initiated for a duration of 8 min, followed by the induction of anesthesia using 2 mg of midazolam, 10 mg of etomidate, 20 μg of sufentanil, and 16 μg of cisatracurium prior to tracheal intubation. Target-controlled infusions of propofol and remifentanil and occasional injections of cisatracurium were used to maintain anesthesia. A lung-protective ventilation approach was utilized, and end-tidal carbon dioxide (P_ET_CO_2_) was maintained at 35 ~ 45 mmHg with tidal volumes 6 ~ 8 mL/kg, PAW < 20 H_2_O, PEEP 5 ~ 8 cmH_2_O, and FiO_2_ 0.3 ~ 0.5 to ensure SpO_2_ > 96%. A blood transfusion machine was connected to stop any unexpected bleeding.

**Figure 2:**
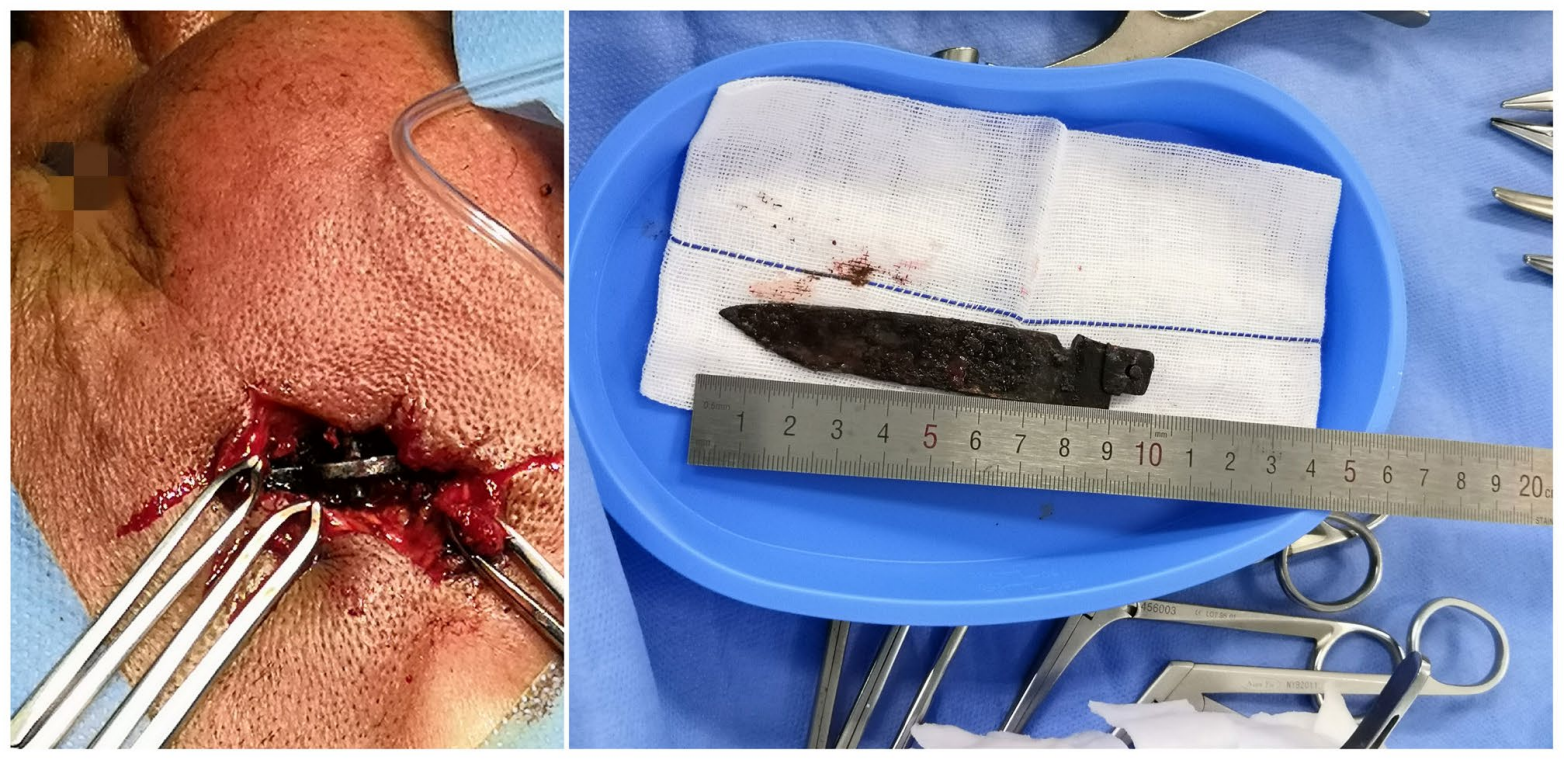
Intracranial foreign body.

The surgical incision was made on the right temporal infected wound. The tissue surrounding the foreign body was separated, and the hilt was located and revealed. The pus was cleared under the nasal endoscope to avoid the interference of a large amount of blood. The surrounding adhesive tissue was loosened, and the precise location of the knife and adjacent tissues was checked. To address local blood oozing and to facilitate smooth drainage through the sphenoid sinus, a hilt of approximately 10 cm was delicately removed from the temporal incision under direct view of the nasal endoscope (Figure 2). The operation was completed successfully after 3 h with no residual foreign body, cerebral hemorrhage, hematoma, or damage to the brain abscess capsule. The patient resumed spontaneous breathing after 10 min, and after 30 min, he was fully awake. After a thorough review, the tracheal tube was removed, and the patient was sent to the neurological intensive care unit (NICU).

**Figure 3:**
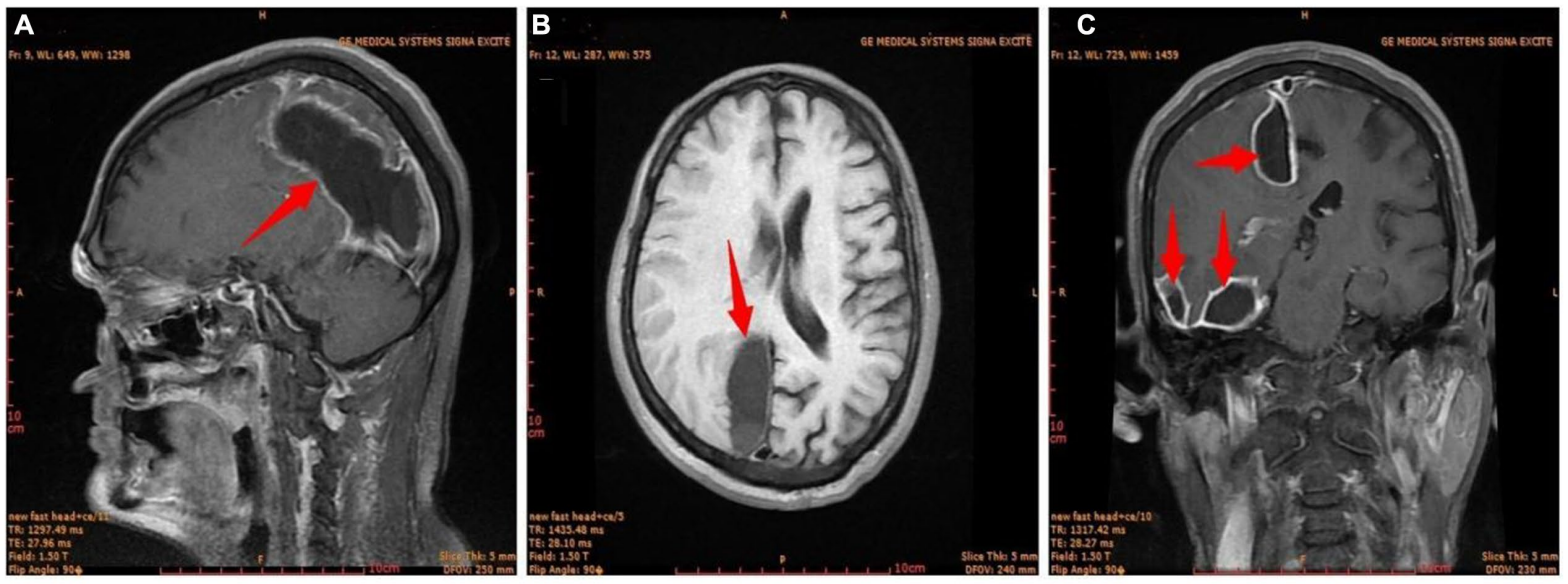
The first postoperative craniocerebral MR image. **(A)** Sagittal; **(B)** transverse; **(C)** coronal. Red arrow, brain abscess.

**Figure 4:**
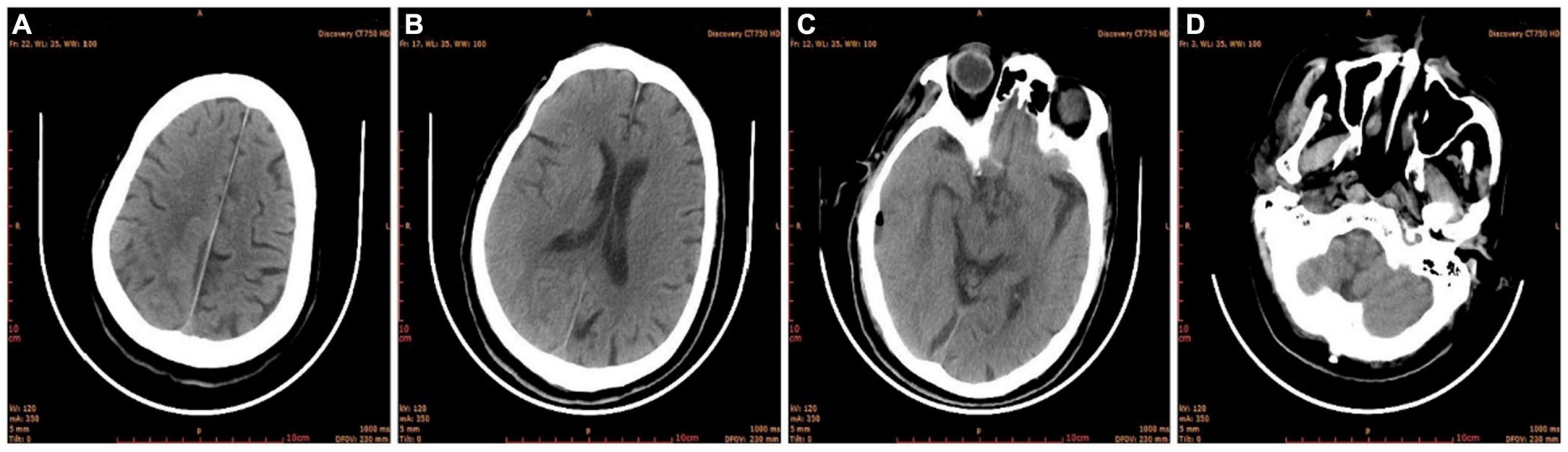
Brain CT before discharge. **(A)** Semi-oval central level; **(B)** lateral ventricular body level; **(C)** cerebral base level; **(D)** skull base level.

The condition of the right eye steadily improved 6 h after the first surgery. Multiple brain abscesses were discovered during postoperative MR examination (Figure 3). On the 3rd postoperative day, the intracranial abscess was punctured and drained under monitored anesthesia care (MAC). Blood and pus samples were subjected to a drug sensitivity test and bacterial culture, respectively. Postoperative recovery went well. After the second surgery, the motor function and muscle strength of the left upper and lower limbs gradually improved. There were no signs of widespread infection. He was discharged from the hospital on April 11 after a CT scan showed no abnormalities (Figure 4).

## Discussion

Areas located above 2,500 m of elevation are called plateau areas in medicine (8). The low air pressure in plateau areas also reduces the oxygen partial pressure, which leads to hypoxia and high-altitude sickness (9). Human alveolar oxygen partial pressure, arterial blood oxygen partial pressure, and oxygen saturation are lower than those in plain areas as a result of the decreased atmospheric oxygen partial pressure (4, 6). It is more challenging to maintain the safety of anesthesia for surgical patients who live in plateau areas (10). Plateau residents have a strong tolerance to hypoxia, and the main problems are physiological changes that take place during high-altitude acclimation, such as an increase in hematocrit, pulmonary hypertension, PaCO_2_, and a decrease in the blood bicarbonate concentration (9, 11, 12). The key to anesthesia management is to maintain the patient’s PaO_2_ and PaCO_2_ at their preoperative baseline, rather than at the conventionally normal level.

Long-term plateau dwellers often exhibit general weakness, vertigo, insomnia, and other unpleasant symptoms when they travel to low-altitude areas for short periods (6, 13, 14). Sedatives can be properly administered to patients with severe symptoms to promote sleep and lessen blood pressure changes. Antihypertensive treatment is administered to patients with hypertension from admission until the morning of the operation. An atropine test is typically conducted before surgery for patients whose heart rate is less than 50 bpm. When an atropine test is negative, thus sick sinus syndrome can be ruled out, anesthesia can be considered (13).

To increase the patient’s oxygen tolerance and decrease their pulmonary vascular resistance and right ventricular afterload, we immediately initiate oxygen inhalation through the nasal catheter, and oxygen and nitrogen removal last more than 8 min during anesthesia induction. Arterial blood gas is assessed in a timely manner to check for respiratory alkalosis. Respiratory alterations, including increased lung ventilation, specifically increased tidal volume, and respiratory rate are a concern at high altitudes in patients under general anesthesia. The respiratory system must remain unobstructed, respiratory secretions must be promptly removed, ventilation must be adequately increased, the concentration of breathed oxygen must be increased, and a small tidal volume lung-protective ventilation approach must be used.

As a result of the elevated altitude, decreased air density, and diminished partial pressure of oxygen, the Haibei Tibetan Autonomous Prefecture at an elevation of 3,200 m exhibits an approximate partial pressure of oxygen of 700 Hpa (equivalent to 60% of that found at sea level). Consequently, the arterial oxygen saturation of long-term residents in this region typically hovers approximately 88%. This reduction in oxygen reserves within the body leads to an elevation in respiratory rate, resulting in a state of respiratory alkalosis. Prior to surgery, the oxygen reserve should increase; the oxygen consumption should decrease; and the oxygen supply should be increased throughout the perioperative period (14). Our approach includes full oxygen mechanical ventilation, preoxygenation for 8 min prior to anesthesia induction, timely blood infusions throughout the procedure, and the application of vasoactive drugs to ensure stable circulation. At the same time, anesthesia, analgesia, and sedation should be adequate to prevent stress reactions from increasing oxygen consumption and exacerbating hypoxia. When using anesthetic drugs, the metabolic rate, oxygen consumption, and circulatory function of the body should be reduced to lessen drug interference. The volume-controlled ventilation mode of lung protection strategy of controlling respiratory frequency and reducing tidal volume was adopted to correct respiratory alkalosis and keep PaCO_2_ at 35 ~ 45 mmHg. Lung-protective ventilation strategy can also effectively prevent the occurrence of mechanical ventilation-related lung injury (15, 16).

In this patient, goal-directed volume-restricted fluid management was utilized in the maintenance of anesthesia for the patient. The preoperative hemoglobin levels of patients in the plateau area were found to be (183 ± 11) g/L, with a range of 140 to 205 g/L. The elevation of hemoglobin levels results in an augmentation of the oxygen-carrying capacity of the blood, leading to an increase in blood viscosity and potentially impacting the transport and exchange of oxygen due to microcirculatory stasis (17). We contended that patient volume should be limited through goal-directed fluid administration, with vasoactive medications utilized to manage hemodynamics and carefully regulate fluid volume. Crystalloid infusion, specifically glucose and Ringer’s solution in a 1:1 ratio (excluding patients with diabetes), served as the primary method of fluid administration. Colloidal solutions, plasma, and concentrated red blood cells were administered based on the presence of bleeding and blood pressure levels during surgical procedures. This intervention serves to restore the patient’s blood volume, stabilize hemodynamics, prevent perioperative thrombosis, and alleviate the strain on vital organs such as the heart, brain, and lungs.

Severe brain injury has been associated with very high rates of mortality and disability. In clinical practice, early identification of mortality risk factors is crucial for managing traumatic brain injury and determining the associated prognosis. The effects of brain injury on the body and the associated prognosis in patients living in plateau areas vary greatly from those in patients living in plain areas due to the uniqueness of the environment (18). Increased cerebrospinal fluid and brain volume are the main pathophysiological causes of acute cerebral coma and intracranial hypertension syndrome (19). The primary goals of perioperative management for patients with craniocerebral trauma are to increase cerebral blood flow and perfusion and prevent subsequent brain injury (20). Cerebral infection and foreign-body penetration were the main causes of brain injury in this patient. Protecting the brain, preventing acute bleeding, and preventing intracranial infections are the main goals of perioperative anesthesia treatment. Anesthesia medications with brain-protective properties (such as propofol and dexmedetomidine) were used because the patient was older than 70 years, and the systolic blood pressure was kept at or above 110 mmHg during the procedure. Antibiotics were used as a preventative measure during surgery to reduce the risk of infection and postoperative pneumonia.

In summary, it is a miracle that this patient survived in such a complex situation for a long time and received effective treatment. Our observations are as follows.

1. The patient experienced only a brief coma and has survived for 26 years. The initial injury did not damage the skull because the penetration path of the knife was near the lateral skull base.
2. As the patient aged, the ability of the body to defend against foreign bodies weakened, thus leading to cerebral infections that impaired neuronal transmission and eyesight.
3. The formation of an abscess around the knife and its separation from the surrounding tissue provided the best opportunity for successful foreign-body extraction.
4. Acute massive bleeding should be actively managed by properly preparing the blood before surgery by using a blood transfusion machine and diluting the blood during surgery.
5. Intraoperative intracranial hypertension was avoided. Lung-protective ventilation strategies were adopted to avoid a series of reactions caused by “oxygen poisoning.”
6. The risk of microthrombosis was reduced.
7. The operation was performed in the compound operating room. Intraoperative CT or MR examination was performed to diagnose emergent adverse events in a timely manner.
8. Preoperative multidisciplinary teamwork, accurate surgical planning, and perioperative management strategies effectively ensure patient safety.

Treatment challenges include diseases particular to the plateau area, severe craniocerebral injury, and advanced age. For anesthesiologists, it is essential to fully understand the patient’s history before surgery, assess the risk of the procedure, and ensure precision in the surgical plan and the management of anesthesia.

## Data availability statement

The original contributions presented in the study are included in the article/supplementary material, further inquiries can be directed to the corresponding authors.

## Ethics statement

Written informed consent was obtained from the individual(s) for the publication of any potentially identifiable images or data included in this article.

## Author contributions

YS: Conceptualization, Funding acquisition, Writing – original draft, Writing – review & editing. YL: Investigation, Writing – original draft. PL: Data curation, Investigation, Writing – original draft. MZ: Conceptualization, Project administration, Supervision, Writing – original draft. ML: Formal analysis, Investigation, Writing – review & editing. YW: Funding acquisition, Writing – original draft, Writing – review & editing.
